# Supplementary material for: Metagenomics of the modern and historical human oral microbiome with phylogenetic studies on Streptococcus mutans and Streptococcus sobrinus
Source: Philos Trans R Soc Lond B Biol Sci. 2020 Oct 5;375(1812):20190573. doi: 10.1098/rstb.2019.0573 (PMC7702799; doi:10.1098/rstb.2019.0573)
Supplement: Table S2. [file rstb20190573supp3.docx]

Philosophical Transactions of the Royal Society B

Metagenomics of the modern and historical human oral microbiome with phylogenetic studies on *Streptococcus mutans* and *Streptococcus sobrinus*

Mark Achtman and Zhemin Zhou

Table S2. Oral samples that were excluded from further analysis because <15% of their microbial reads were from the 50 most common oral taxa.

| **Sample** | **Source** | **MBases** | **Center Name** |
| --- | --- | --- | --- |
| EBC2 | dental calculus | 17 | OAGR, University of Adelaide |
| EBC3 | dental calculus | 60 | OAGR, University of Adelaide |
| Spy2 | dental calculus | 288 | OAGR, University of Adelaide |
| Sudan1 | dental calculus | 11 | OAGR, University of Adelaide |
| Sudan2 | dental calculus | 27 | OAGR, University of Adelaide |
| SRS943643 | saliva | 10,490 | BIOLS |
| SRS943644 | saliva | 3,820 | BIOLS |
