# Supplementary material for: Metagenomics of the modern and historical human oral microbiome with phylogenetic studies on Streptococcus mutans and Streptococcus sobrinus
Source: Philos Trans R Soc Lond B Biol Sci. 2020 Oct 5;375(1812):20190573. doi: 10.1098/rstb.2019.0573 (PMC7702799; doi:10.1098/rstb.2019.0573)
Supplement: Figure S1. [file rstb20190573supp8.pdf]

Metagenomics of the modern and historical human oral microbiome with phylogenetic studies on *Streptococcus mutans* and *Streptococcus sobrinus*

Mark Achtman and Zhemin Zhou

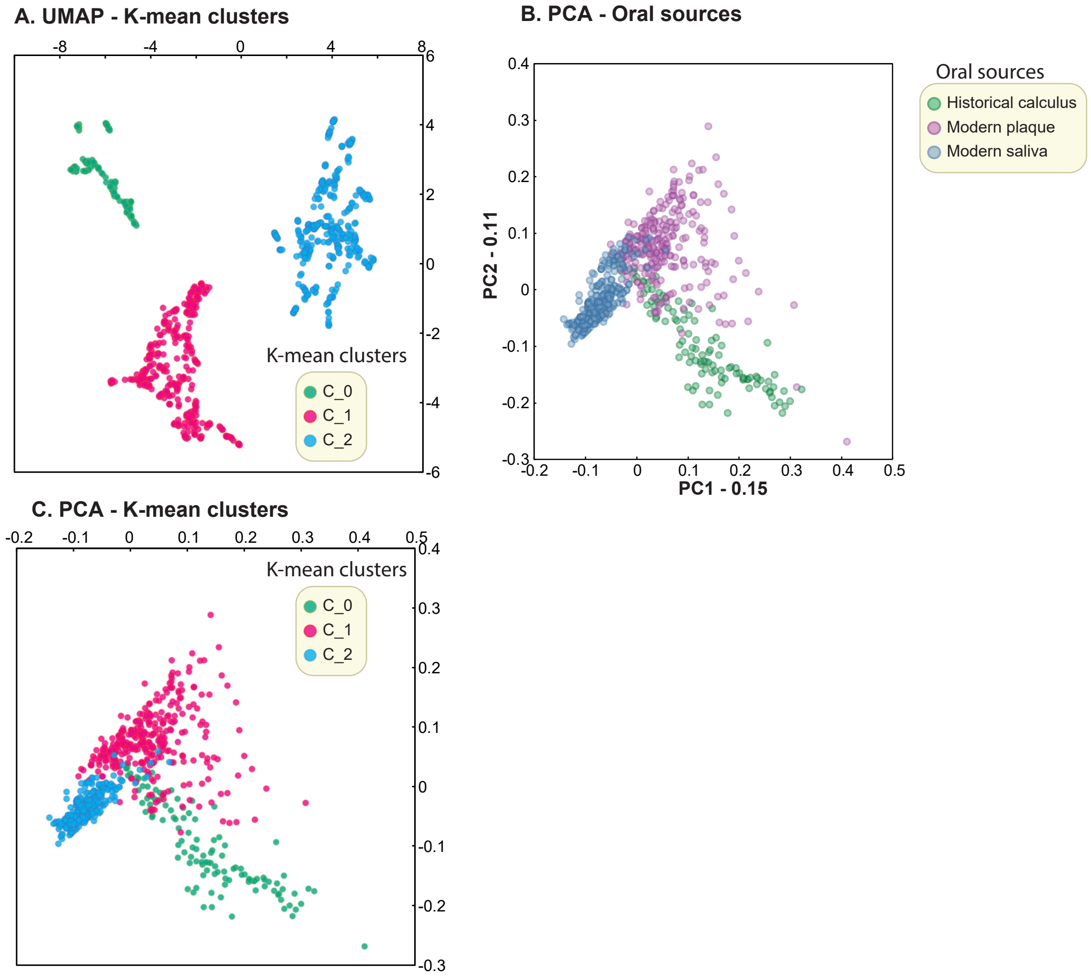

Figure S1. The K-mean clusters of the first three components of the UMAP analyses and their visualization in PCA plots. (A) The visualization of the three clusters in the plane of UMAP components. (B) Plot of the first two components of the PCA analysis. (C) The same plot as (B) with nodes color-coded by K-mean clusters in (A).
