# Supplementary material for: Metagenomics of the modern and historical human oral microbiome with phylogenetic studies on Streptococcus mutans and Streptococcus sobrinus
Source: Philos Trans R Soc Lond B Biol Sci. 2020 Oct 5;375(1812):20190573. doi: 10.1098/rstb.2019.0573 (PMC7702799; doi:10.1098/rstb.2019.0573)
Supplement: Figure S3. [file rstb20190573supp10.pdf]

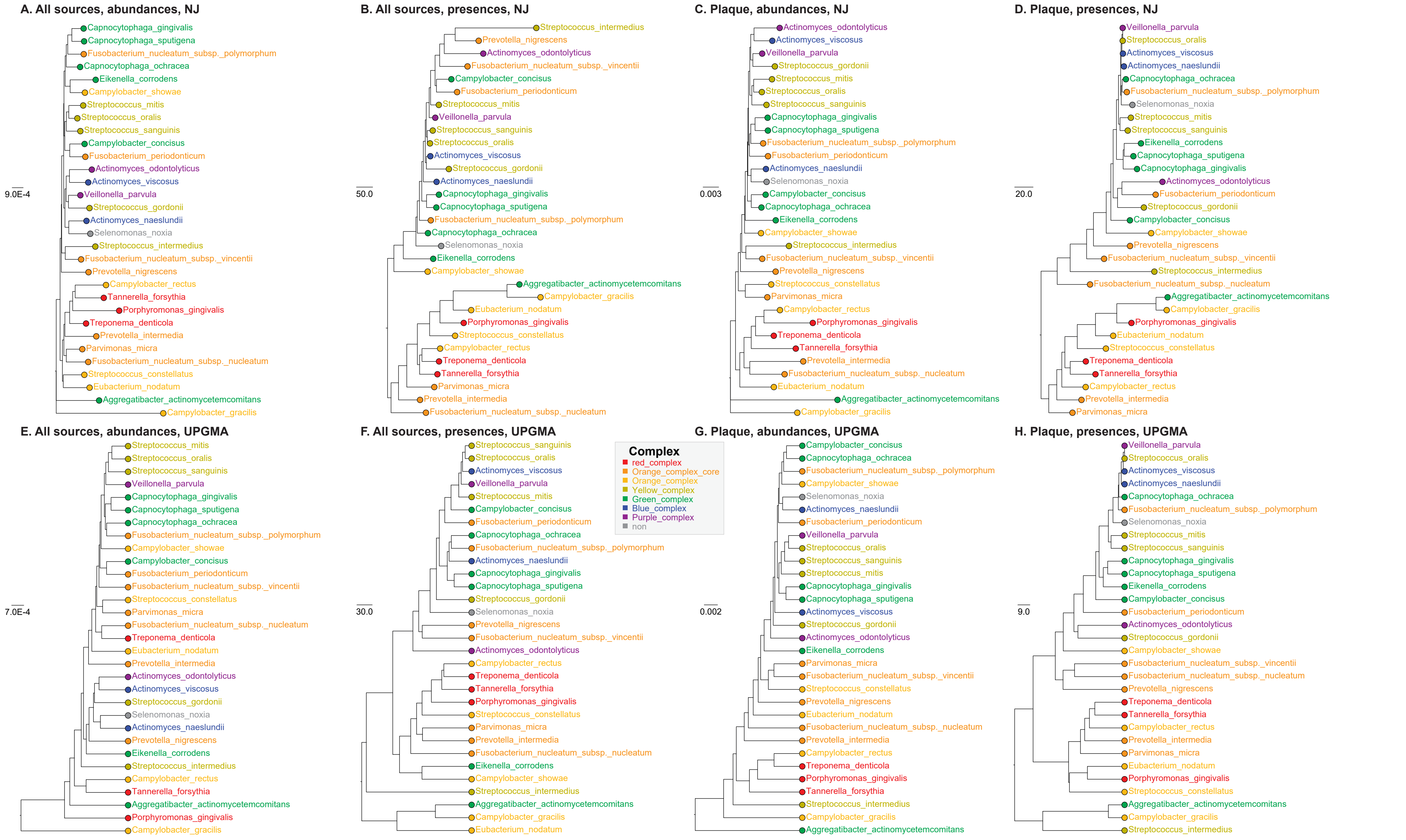

Figure S3. Neighbor-joining and UPGMA clustering of the 28 species described in Socransky et al. based on their abundances or presences in the oral samples.
