# Supplementary material for: Metagenomics of the modern and historical human oral microbiome with phylogenetic studies on Streptococcus mutans and Streptococcus sobrinus
Source: Philos Trans R Soc Lond B Biol Sci. 2020 Oct 5;375(1812):20190573. doi: 10.1098/rstb.2019.0573 (PMC7702799; doi:10.1098/rstb.2019.0573)
Supplement: Figure S4. [file rstb20190573supp11.pdf]

Philosophical Transactions of the Royal Society B

Metagenomics of the modern and historical human oral microbiome with phylogenetic studies on *Streptococcus mutans* and *Streptococcus sobrinus*

Mark Achtman and Zhemin Zhou

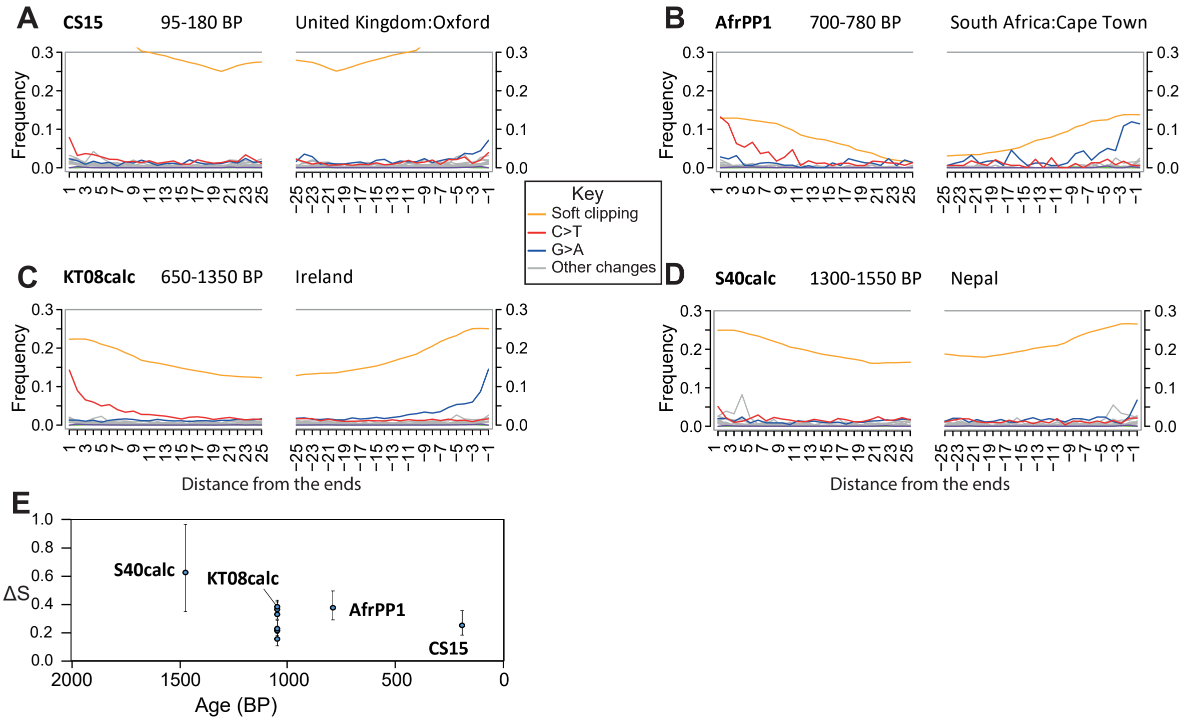

Figure S4. DNA damage at the 5′-end of *S. mutans* sequencing reads from ancient metagenomes. (A-D) MapDamage2 [49] analyses on BAM alignments calculated by Minimap2 [37] of species-specific reads from four selected historical samples against the *S. mutans* UA159 reference genome. Numbers of specific reads were A: 6224; B: 910; C: 26,333; D: 7379. (E) Average MapDamage2 estimates (Δ_S) plus 95% confidence intervals for all ancient metagenomes in which at least 10 *S. mutans* reads were present and at a frequency of ≥0.0001% of all metagenomic reads.
