# Supplementary figures and images for: Metagenomics of the modern and historical human oral microbiome with phylogenetic studies on Streptococcus mutans and Streptococcus sobrinus
Source: Philos Trans R Soc Lond B Biol Sci. 2020 Oct 5;375(1812):20190573. doi: 10.1098/rstb.2019.0573 (PMC7702799; doi:10.1098/rstb.2019.0573)

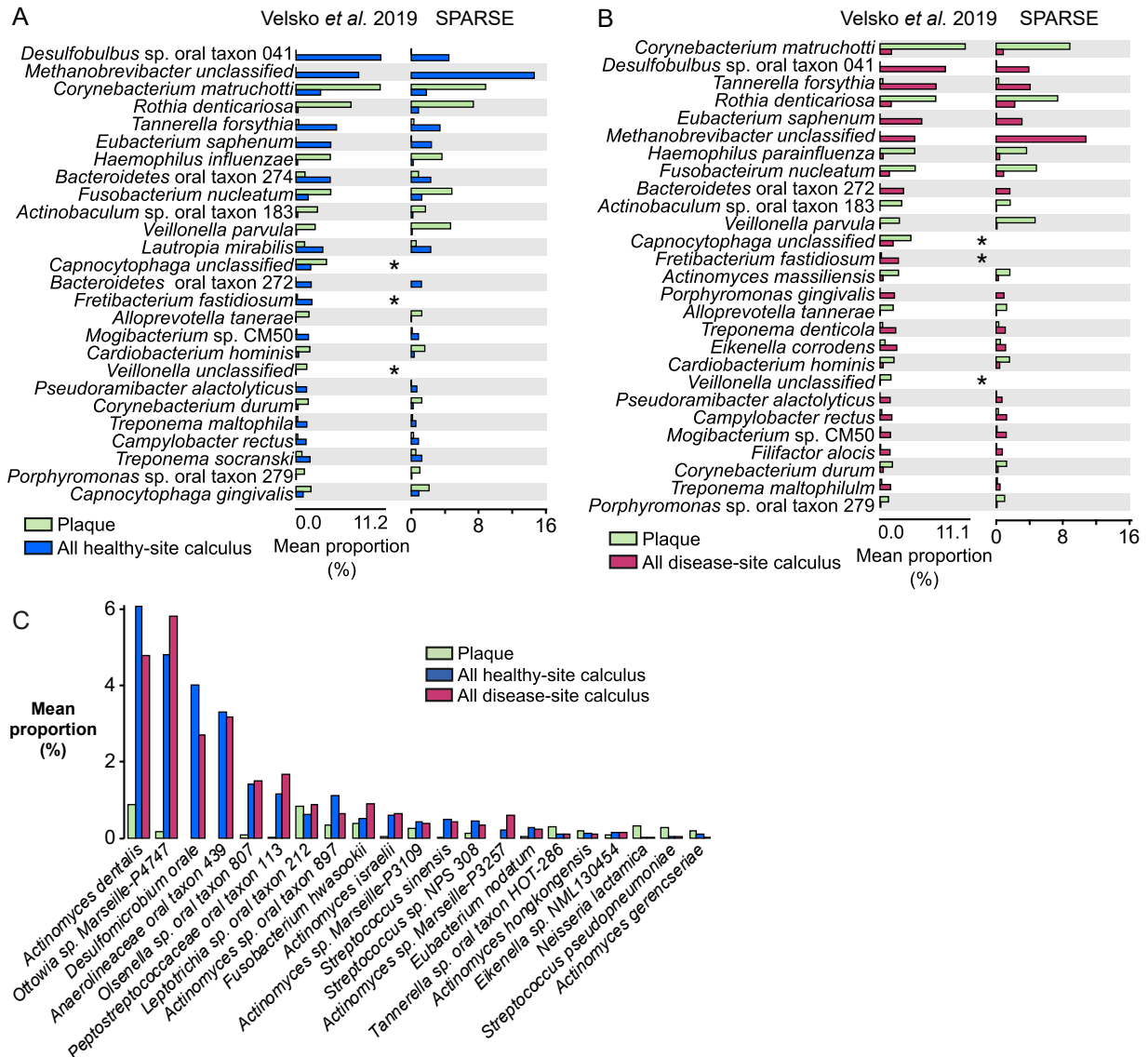

Supplement: Figure S7. [file rstb20190573supp14.pdf]
