# Supplementary material for: Metagenomics of the modern and historical human oral microbiome with phylogenetic studies on Streptococcus mutans and Streptococcus sobrinus
Source: Philos Trans R Soc Lond B Biol Sci. 2020 Oct 5;375(1812):20190573. doi: 10.1098/rstb.2019.0573 (PMC7702799; doi:10.1098/rstb.2019.0573)
Supplement: Figure S9. [file rstb20190573supp16.pdf]

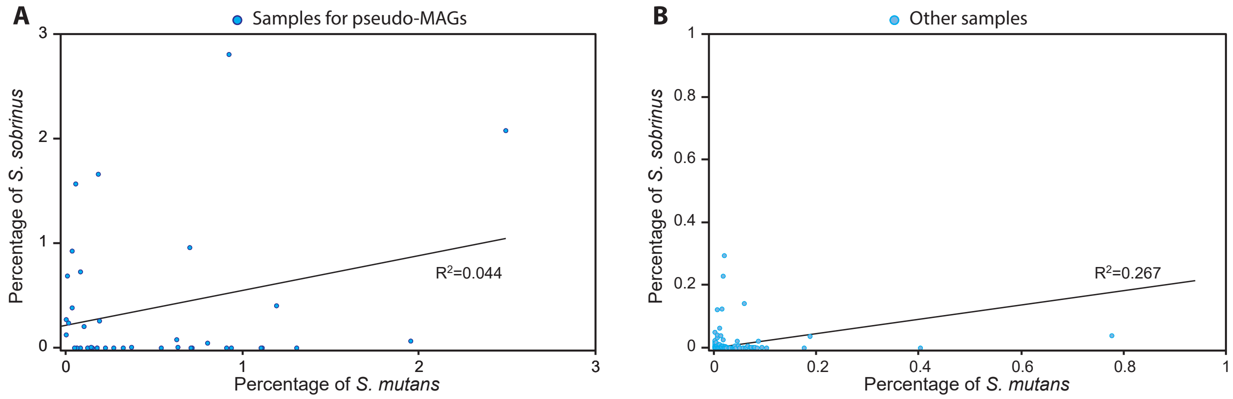

Figure S9. Abundances of *S. mutans* (X axis) and *S. sobrinus* (Y axis) in the modern metagenomes that were used to reconstruct pseudo-MAGs (A) and from the low numbers of reads in all other samples (B). The linear regressions (black lines) indicate low correlations between the co-abundances of the two species.
