# Supplementary material for: Metagenomics of the modern and historical human oral microbiome with phylogenetic studies on Streptococcus mutans and Streptococcus sobrinus
Source: Philos Trans R Soc Lond B Biol Sci. 2020 Oct 5;375(1812):20190573. doi: 10.1098/rstb.2019.0573 (PMC7702799; doi:10.1098/rstb.2019.0573)
Supplement: Figure S2. [file rstb20190573supp9.pdf]

Philosophical Transactions of the Royal Society B

Metagenomics of the modern and historical human oral microbiome with phylogenetic studies on *Streptococcus mutans* and *Streptococcus sobrinus*

Mark Achtman and Zhemin Zhou

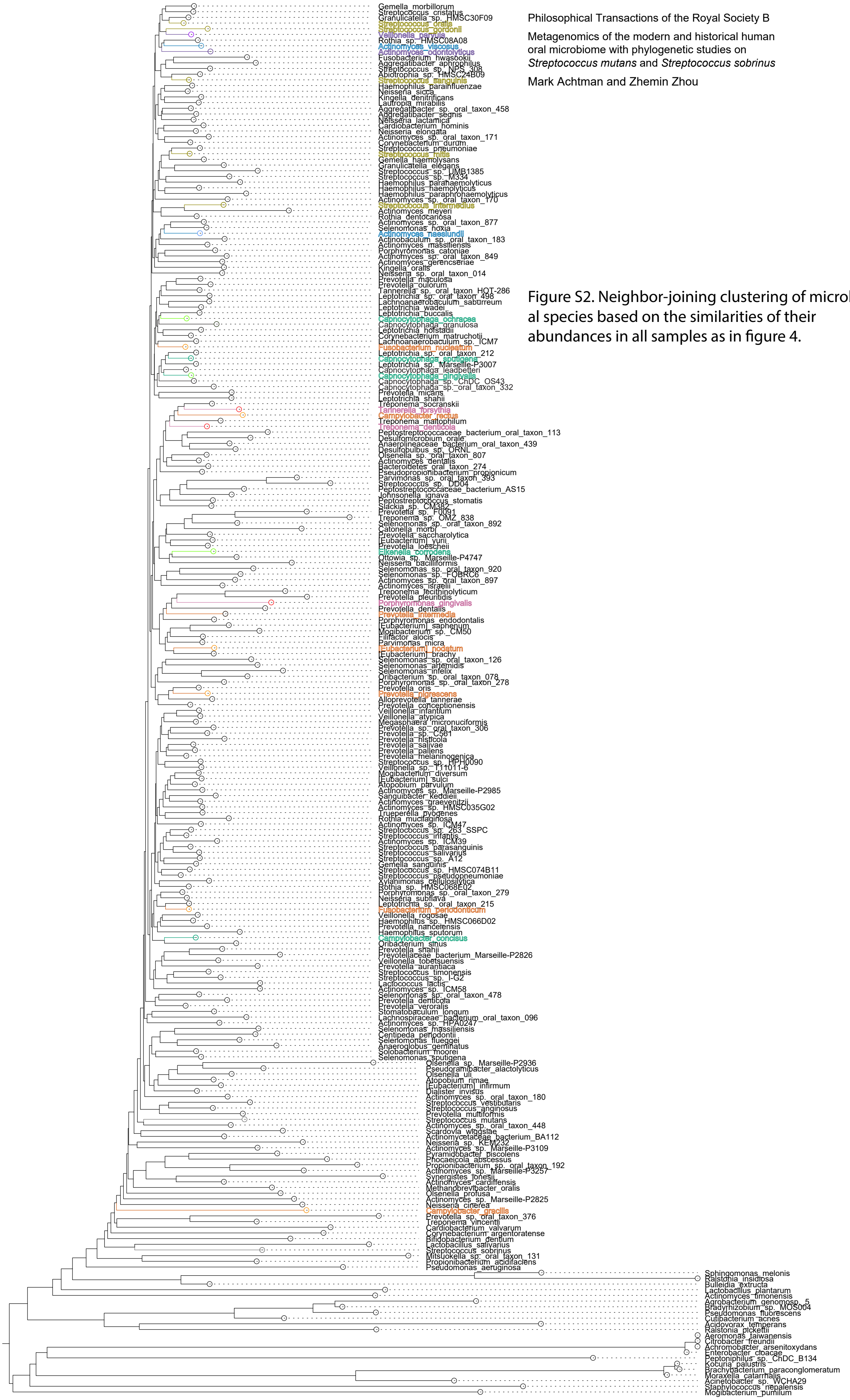

Figure S2. Neighbor-joining clustering of microbial species based on the similarities of their abundances in all samples as in figure 4.
